# Relationship Between Negative Emotions and Job Burnout in Medical Staff During the Prevention and Control of the COVID-19 Epidemic: The Mediating Role of Psychological Resilience

**DOI:** 10.3389/fpsyt.2022.857134

**Published:** 2022-06-22

**Authors:** Yao Chen, Libin Zhang, Huan Qi, Wei You, Chencong Nie, Li Ye, Ping Xu

**Affiliations:** ^1^Emergency Department, Zigong Fourth People's Hospital, Zigong, China; ^2^Collaborative Innovation Center of Assessment for Basic Education Quality, Beijing Normal University, Beijing, China; ^3^School of Psychology and Mental Health, North China University of Science and Technology, Tangshan, China; ^4^Emergency Department, Fushun People's Hospital, Fushun, China

**Keywords:** COVID-19, psychological resilience, negative emotion, job burnout, mediating role

## Abstract

We herein investigated the relationship between psychological status and the various emotions of medical staff during the prevention and control of coronavirus disease 2019 (COVID-19) epidemic. In this study, the convenience sampling method was used to select medical staff members as participants, and a cross-sectional study design was implemented. The instruments included the Burnout Clinical Subtype Questionnaire (BCSQ-36), the 10-item Connor-Davidson Resilience Scale (CD-RISC-10), the self-rated 16-item Quick Inventory of Depressive Symptomatology (QIDS-SR16), and the Self-rating Anxiety Scale (SAS). In total, 876 medical staff members were selected in this study. The CD-RISC-10 was negatively correlated with all other scales (*P* < 0.01). The hierarchical regression coefficients of the SAS and QIDS-SR16 against the BCSQ-36 mediated by the CD-RISC-10 were *P* < 0.01, and the significance of the F values in all hierarchical regression equations was P < 0.01 (Sobel test, *P* < 0.01). Medical staff burnout during the COVID-19 epidemic was affected by anxiety and depression, and psychological resilience had a mediating role. Attending to changes in the negative emotions of medical staff and improving their psychological resilience are beneficial to alleviate job burnout.

## Introduction

The global pandemic of coronavirus disease 2019 (COVID-19) has caused a public health emergency since its outbreak (1). COVID-19 has caused and aggravated many mental health problems in different groups of people. Several studies have reported symptoms of anxiety and depression in healthcare workers (2). Some medical personnel have experienced emotional exhaustion, which affects their physical and mental health and may compromise their productivity and quality of healthcare administered (3). A better understanding of mental health of medical staff during the prevention and control of COVID-19 is urgently required.

Emotional exhaustion is a major cause of medical staff members' negative emotions (3). A cross-sectional study reported a high prevalence of depression, anxiety, and post-traumatic stress symptoms among healthcare workers during the COVID-19 outbreak (4). These negative emotional states can cause individuals to experience a reduced sense of accomplishment and burnout in their work and studies.

Burnout is a syndrome caused by medium- and long-term stress at work. Studies in China and abroad have reported high risk for job burnout in medical staff attending to COVID-19 patients (5, 6). Furthermore, because of increased work pressure and vulnerability to depression and post-traumatic stress disorder during COVID-19, job burnout was common among healthcare workers (7). Medical personnel form the backbone of the prevention and control of the COVID-19 epidemic, and according to previous studies, they were under great psychological pressure because of COVID-19, thus increasing the rate of job burnout (8).

Psychological resilience immensely helps individuals with responding and adapting to setbacks. It is an important psychological resource for improving emotional adjustment and can effectively relieve psychological stress (9). A previous study revealed that psychological resilience was beneficial for reducing negative emotions, such as anxiety, during the COVID-19 epidemic (10). Psychological resilience reportedly acts as a determinant of positive effects on burnout (11). It has been shown that the mediator of psychological resilience between negative emotions and burnout of female nurses (12).

This study aims to estimate negative emotions, job burnout, and psychological resilience in medical staff during COVID-19 and to present the relationships among the three psychological factors. This can help identify ways to promote the mental health of medical staff and can provide theoretical bases for improving emergency response training for medical personnel. The present exploratory study hypothesizes that psychological resilience buffers the effects of negative emotions in healthcare workers experiencing job burnout.

## Methods

### Participants

In this study, we used the convenience sampling method to select medical staff from 31 medical care institutions of all levels in Sichuan Province, China, as participants from March 1, 2020, to March 31, 2020. We invited 31 volunteers from 31 hospitals to investigate their medical staff. A professional questionnaire survey platform called “Wenjuan Xing” was used to collect information on demographic characteristics of medical staff and collect the response data from the questionnaires administered to medical staff via 85 WeChat groups. The inclusion criteria were as follows: (1) first-line clinical medical staff who were qualified to practice and (2) medical staff who signed informed consent forms and agreed to participate in this study. The study excluded participants with confirmed mental illnesses.

### Procedures

A total of 876 questionnaires were distributed in this study, and the response rate was 100%. The responded questionnaires were carefully reviewed and screened. A pre-survey estimation of the time needed to completely answer the questionnaire revealed 300 s as the minimum time needed to answer the questionnaire. Therefore, questionnaires with a completion time of <300 s were excluded; furthermore, questionnaires with the same answer selected for all items were excluded as well. A total of 35 (4%) questionnaires were excluded on these grounds, and the remaining 841 (96%) questionnaires were considered valid. After 2 weeks of administering the large-sample survey, 200 questionnaires in the database were randomly selected for re-testing. A total of 194 copies were effectively recovered in the re-test, and the effective recovery rate was 97%.

### Instruments

#### Burnout Clinical Subtype Questionnaire (BCSQ-36)

The Burnout Clinical Subtype Questionnaire (BCSQ)-36 is a burnout measurement tool developed by Montero-Marín (13). It has shown relatively good reliability and validity in studies conducted in other countries. This scale contains 36 items and is divided into three subscales: frenetic, underchallenged, and worn-out. Each subscale uses a 7-point Likert scale; each item is scored from 1–7, where 1 = totally disagree and 7 = totally agree. Higher scores indicate higher burnout levels. The BCSQ-36 scale has been widely applied in other countries (14). In this study, the Cronbach's α coefficient of this scale was 0.904. For the three subscales, Cronbach's α coefficients were 0.863–0.925, and the re-test Cronbach's α coefficients were 0.863–0.922. These results indicate that this Chinese BCSQ-36 scale had excellent reliability.

#### Ten-Item Connor–Davidson Resilience Scale (CD-RISC-10)

The Connor–Davidson Resilience Scale (CD-RISC)-10 is a self-reported scale comprising 10 items aimed at measuring mental resilience; this scale is based on the 25-item CD-RISC by Connor et al. (15). Each item on the CD-RISC-10 is scored from 0 to 4 points, and the total score is 0–40 points. Higher scores indicate better psychological resilience (16). This scale has shown high reliability and validity in studies in China (17). In this study, the Cronbach's α coefficient of this scale was 0.953.

#### Self-Rated 16-Item Quick Inventory of Depressive Symptomatology–Self-Report (QIDS-SR16)

The 16-item Quick Inventory of Depressive Symptomatology–Self-Report (QIDS-SR16) questionnaire evaluates residual symptoms and their severity in surveyed subjects. It comprises a total of 16 items with 4-level scoring from 0 to 3 points. Higher scores indicate more severe depression symptoms (18). This scale has been extensively used in China (19). In this study, the Cronbach's α coefficient of this scale was 0.780.

#### Self-Rating Anxiety Scale (SAS)

The Self-rating Anxiety Scale (SAS) is a classic psychological self-rating scale. Higher scores indicate more severe anxiety symptoms (20). In this study, the Cronbach's α coefficient of this scale was 0.677.

### Statistical Analysis

The questionnaire-based survey was administered, and Excel data were exported from the server for statistical analysis using SPSS 25.0. Categorical variables were described by absolute and relative frequencies. Quantitative normally distributed variables were described by the mean and the respective standard deviation (SD). Quantitative non-normally distributed variables were described by the median (Mdn) and the respective interquartile interval (Q1; Q3). The normality of distributions was verified by observation of the respective histograms. To decide which independent variables to include in each multiple regression, simple linear regressions were performed with each of the following variables: hhospital level (Level 1; Level 2; Level 3), sex, age (≤20 years; 20–30 years; 31–40 years; 41–50 years; >50 years), highest education level (associate degree and below; undergraduate degree; master's degree and above), technical title (none; primary; intermediate; advanced), doctor/nurse (doctor; nurse), marital status (unmarried; married;other). All variables that correlated with the outcomes at *p* ≤ 0.20 in a simple regression were included in the multiple linear regressions. The Spearman's analysis was performed for the correlation analysis among BCSQ-36, CD-RISC-10, QIDS-SR16, and SAS. A hierarchical regression model was estimated to examine the mediating role of resilience in the relationship between depression or anxiety and job burnout. The following requirements for such analysis were verified: a significant correlation between depression or anxiety (independent variable) and job burnout (dependent variable); a significant correlation between depression or anxiety and resilience (the mediator) and between resilience and job burnout. Additionally, the effect of depression or anxiety on job burnout should shrink (partial mediator) or become statistically insignificant (full mediator) after the inclusion of resilience in the model. Standardized estimates (β), F statistics, determination coefficient (R2), and R2 -changes (ΔR2) for each step were provided. Multicollinearity was checked through tolerances and variance inflation factors ranges. Finally, the Sobel test was pursued to assess the mediation effect.

Values of *p* ≤ 0.05 were considered significant.

### Demographic Characteristics

The survey responses to the demographic characteristics and scores for job burnout, resilience, depression, and anxiety are presented in Table 1. A total of 841 doctors and nurses completed the study questionnaire. The majority of the total participants (96.2%) were from tertiary hospitals. Furthermore, 54.9% of them were aged 20–30 years. In addition, 83.9% of medical staff comprised nurses, and 60.4% of them had completed an undergraduate degree.

**Table 1:** General information of the study subjects (*n* = 841).

	**Demographic variables**	***n* (%)**	**BCSQ-36**	***P***	**CD-RISC-10**	***P***	**QIDS-SR16**	***P***	**SAS**	***P***
Hospital level	Level 3	809 (96.2)	131.0 (36.0,246.0)	0.079	39.0 (10.0,50.0)	0.052	22.5 (17.0,37.0)	0.277	29.5 (24.0,47.0)	0.556
	Level 2	19 (2.3)	136.0 (43.0,158.0)		37.0 (10.0,50.0)		23.0 (16.0,35.0)		32.0 (23.0,44.0)	
	Level 1	13 (1.5)	136.5 (115.0,167.0)		35.0 (22.0,41.0)		21.0 (16.0,64.0)		32.0 (20.0,67.0)	
Sex	Male	138 (16.4)	130.0 (36.0,246.0)	0.005	40.0 (10.0,50.0)	0.030	20.0 (16.0,64.0)	0.068	31.0 (22.0,67.0)	0.494
	Female	703 (83.6)	132.0 (51.0,180.0)		38.0 (10.0,50.0)		21.0 (16.0,44.0)		32.0 (20.0,51.0)	
Age, years	20~30	462 (54.9)	131.0 (38.0,180.0)	0.636	37.5 (10.0,50.0)	0.000	21.0 (16.0,44.0)	0.422	32.0 (20.0,51.0)	0.428
	31~40	267 (31.7)	133.0 (51.0,176.0)		39.0 (10.0,50.0)		22.0 (16.0,42.0)		32.0 (20.0,45.0)	
	41~50	87 (10.3)	130.0 (36.0,246.0)		40.0 (10.0,50.0)		22.0 (16.0,64.0)		33.0 (22.0,67.0)	
	>50	25 (3.0)	128.0 (85.0,149.0)		44.0 (33.0,50.0)		20.0 (16.0,32.0)		31.0 (25.0,42.0)	
Highest education level	Associate degree and below	313 (37.2)	127.5 (96.0,153.0)	0.963	37.5 (22.0,46.0)	0.664	22.0 (18.0,39.0)	0.195	31.0 (21.0,46.0)	0.855
	Undergraduate degree	508 (60.4)	130.0 (63.0,178.0)		38.0 (10.0,50.0)		21.0 (16.0,44.0)		32.0 (20.0,51.0)	
	Master's degree and above	20 (2.4)	132.0 (36.0,246.0)		39.0 (10.0,50.0)		22.0 (16.0,64.0)		32.0 (20.0,67.0)	
Technical title	None	196 (23.3)	130.0 (64.0,178.0)	0.013	38.0 (10.0,50.0)	0.002	21.0 (16.0,44.0)	0.572	32.0 (20.0,51.0)	0.175
	Primary	365 (43.4)	131.0 (38.0,180.0)		38.0 (10.0,50.0)		22.0 (16.0,41.0)		32.0 (20.0,49.0)	
	Intermediate	193 (22.9)	133.0 (51.0,174.0)		39.0 (10.0,50.0)		21.0 (16.0,46.0)		32.0 (20.0,46.0)	
	Advanced	87 (10.4)	131.0 (36.0,246.0)		40.0 (10.0,50.0)		22.5 (17.0,37.0)		33.0 (20.0,67.0)	
Doctor/ nurse	Doctor	135 (16.1)	131.0 (36.0,246.0)	0.016	40.0 (10.0,50.0)	0.001	21.0 (16.0,64.0)	0.642	32.0 (20.0,67.0)	0.097
	Nurse	706 (83.9)	131.0 (38.0,180.0)		38.0 (10.0,50.0)		21.0 (16.0,46.0)		32.0 (20.0,51.0)	
Marital status	Unmarried	259 (30.8)	130.0 (43.0,180.0)	0.627	38.0 (10.0,50.0)	0.167	21.0 (16.0,44.0)	0.030	31.0 (20.0,49.0)	0.004
	Married	553 (65.8)	131.0 (36.0,246.0)		39.0 (10.0,50.0)		22.0 (16.0,64.0)		32.0 (20.0,67.0)	
	Other	29 (3.4)	131.0 (54.0,168.0)		38.0 (10.0,50.0)		23.0 (16.0,38.0)		34.0 (24.0,51.0)	

First, sex (*P* = 0.005) and nurses (*P* = 0.016) were associated with higher scores of job burnout. Second, younger medical staff (*P* = 0.001), sex (*P* = 0.030), and nurses (*P* = 0.001) were associated with lower scores of CD-RISC-10. Finally, married medical staff was associated with higher depression (*P* = 0.030) and anxiety scores (*P* = 0.004).

### Correlation Analysis of BCSQ-36, CD-RISC-10, QIDS-SR16, and SAS

The BCSQ-36, CD-RISC-10, QIDS-SR16, and SAS results based on the Kolmogorov–Smirnov test all showed *P* < 0.05. The Spearman correlation analysis results showed that CD-RISC-10 had a significantly negative correlation with other variables, *P* < 0.01. There were positive correlations among the other variables. The correlation among all variables was significant, *P* <0.01. The correlation coefficients among all variables are shown in Table 2.

**Table 2:** Spearman correlation analysis of job burnout, psychological resilience, depression, and anxiety.

**Variable**	**Mdn (Q1; Q3)**	**1**	**2**	**3**	**4**	**5**	**6**	**7**
1 BCSQ-36	131.0(36.0,246.0)	1						
2 CD-RISC-10	39.0(10.0,50.0)	−0.415**	1					
3 QIDS-SR16	21.0(16.0,64.0)	0.351**	−0.292**	1				
4 SAS	32.0(20.0,67.0)	0.239**	−0.146**	0.451**	1			
5 Frenetic	55.0(12.0,84.0)	0.485**	−0.217**	0.022**	0.101**	1		
6 Underchallenged	37.0(12.0,80.0)	0.872**	−0.337**	0.354**	0.247**	0.183**	1	
7 Worn out	39.0(12.0,84.0)	0.836**	−0.395**	0.422**	0.335**	0.125**	0.720**	1

**P < 0.01.

### Collinearity Diagnostics

The correlation coefficients between the BCSQ-36 results and the underchallenged and worn-out subscales were above 0.8. Collinearity diagnostics were performed with relevant variables. The variance inflation factor (VIF) value between the BCQS-36 and the underchallenged subscale was 2.351, and the VIF value between the BCQS-36 and the worn-out subscale was 2.558. Therefore, there was no collinearity among the variables.

### Common Method Bias Test

Due to the large number of items in this study, a common method bias test of the research results was conducted. The cumulative variance contribution rate of the first common factor extracted using the exploratory factor analysis was 21.7%. As this value is lower than 40%, this study was identified as not having common method bias.

### Mediating Effect Analysis

#### The Mediating Role of Psychological Resilience in The Relationship Between Anxiety and Job Burnout

To avoid the presence of the intercept terms that were irrelevant to the method in the regression equation, all variables were standardized for hierarchical regression analysis. The mediating role of psychological resilience in the relationship between anxiety and job burnout (outcome) was explored using hierarchical linear regression analyses, and the results are shown in Table 3. The model comprises three steps: in the first step, all of the independent variables considered associated with job burnout were adjusted in a multiple linear regression model (hospital level, highest education level, technical title and marital status); in the following two steps, anxiety was entered and then psychological resilience was included. The obtained model showed a positive association between job burnout and anxiety, explaining 14.0% of job burnout data variance (β = 0.365, *P* < 0.001), and a negative association between job burnout and psychological resilience, accounting for an increase of 19.5% in the explained variance (β = −0.192, *P* < 0.001).

**Table 3:** Analysis of the mediating effect of psychological resilience on influence of anxiety on job burnout.

**Variables**	**Step 1(β)**	**Step 2(β)**	**Step 3(β)**
**Hospital level**			
Level 1		Reference	
Level 2	−0.072	−0.080	−0.073
Level 3	−0.126^*^	−0.146^*^	−0.129^*^
**Highest education level**			
Associate degree and below		Reference	
Undergraduate degree	0.048	0.079	0.085
Master's degree and above	0.122	0.161	0.161
**Technical title**			
None		Reference	
Primary	0.008	0.001	0.092
Intermediate	**-**0.006	−0.002	−0.004
Advanced	0.012	−0.009	0.012
**Marital status**			
Unmarried		Reference	
Married	0.022	−0.014	0.007
Other	0.013	−0.019	−0.006
SAS		0.365^**^	0.342^**^
**CD-RISC-10**			−0.194^**^
*F*	1.270	12.889^**^	36.621^**^
*R* ^2^	0.015	0.146	0.182
Δ*R*^2^	0.003	0.135	0.170

^*^P < 0.05;

^**^P < 0.01.

**Figure 1:**
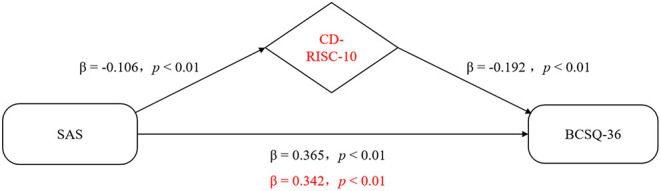
Representative scheme of the mediating role of resilience in the relationship between anxiety and job burnout. Changes in beta weights when the mediator is present are highlighted in red.

Considering that the absolute value of the anxiety's standardized regression coefficient (β) reduced from 0.365 to 0.342 after incorporating psychological resilience into the model (Sobel test, z = 2.741, *P* < 0.001), psychological resilience was found to play a partial mediating role in the association between anxiety and job burnout (Figure 1).

#### The Mediating Role of Psychological Resilience in the Relationship Between Depression and Job Burnout

To avoid the presence of the intercept terms that are irrelevant to the method in the regression equation, all variables were standardized for hierarchical regression analysis. The mediating role of psychological resilience in the relationship between depression and job burnout (outcome) was explored through hierarchical linear regression analyses, and the results are shown in Table 4. The model comprises three steps: in the first step, all of the independent variables considered associated with job burnout were adjusted in a multiple linear regression model (hospital level, highest education level, technical title and marital status); in the following two steps, depression was entered and then psychological resilience was included. The obtained model showed a positive association between job burnout and depression, explaining 17.8% of job burnout data variance (β = 0.412, *P* < 0.001), and a negative association between job burnout and psychological resilience, accounting for an increase of 19.5% in the explained variance (β = −0.140, *P* < 0.001).

**Table 4:** Analysis of the mediating effect of psychological resilience on the influence of depression on job burnout.

**Variables**	**Step 1(β)**	**Step 2(β)**	**Step 3(β)**
**Hospital level**			
Level 1		Reference	
Level 2	−0.072	−0.064	−0.060
Level 3	−0.126^*^	−0.108^*^	−0.098^*^
**Highest education level**			
Associate degree and below		Reference	
Undergraduate degree	0.048	0.113	0.113
Master's degree and above	0.122	0.176	0.173
**Technical title**			
None		Reference	
Primary	0.008	0.002	−0.002
Intermediate	−0.006	0.012	0.021
Advanced	0.012	0.017	0.028
**Marital status**			
Unmarried		Reference	
Married	0.022	0.001	0.007
Other	0.013	−0.027	−0.025
QIDS-SR16		0.415^**^	0.381^**^
**CD-RISC-10**			−0.143^**^
*F*	1.270	172.585^**^	19.565^**^
*R* ^2^	0.015	0.185	0.204
Δ*R*^2^	0.003	0.174	0.192

^*^P < 0.05;

^**^P < 0.01.

**Figure 2:**
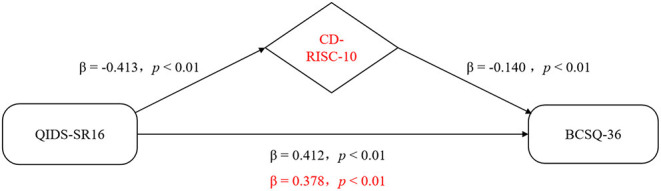
Representative scheme of the mediating role of resilience in the relationship between depression and job burnout. Changes in beta weights when the mediator is present are highlighted in red.

Considering that the absolute value of the depression's standardized regression coefficient (β) reduced from 0.412 to 0.378 after incorporating psychological resilience into the model (Sobel test, z = 3.703, *P* < 0.001), psychological resilience was found to play a partial mediating role in the association between depression and job burnout (Figure 2).

## Discussion

This study investigated various emotions and psychological states and their relationship in medical staff during the prevention and control of COVID-19. The results showed that the four types of psychological states, namely job burnout, psychological resilience, depression, and anxiety, were all significantly correlated with each other (*P* < 0.01). These results indicate that we should pay attention to the interactions among different psychological states instead of considering each psychological state in isolation. In the two first-layer regression models, the regression coefficients of the effect of two negative emotions—anxiety and depression—on burnout were 0.360 and 0.413, respectively (both, *P* < 0.01). Anxiety and depression were identified as independent factors influencing job burnout. The structural equation model showed that the direct effect on job burnout and the total effect after incorporating the mediating effect of negative emotion exogenous latent variables constructed using anxiety and depression as observation variables had a *P* value of <0.05. These results indicate that anxiety and depression had different predictive effects on the job burnout state of medical staff, which corroborates the findings of Vasconcelos et al. (21). Serrão C'S mediational models obtained show that resilience seems to partially mediate the relationships amongst depression and all dimensions of burnout (personal, work-, and client-related burnout) (22). We believe that relieving anxiety and depression in medical staff during the epidemic will alleviate their job burnout state by varying degrees. Strengthening their COVID-19-related training, providing regular information about the state of the COVID-19 epidemic, and increasing medical personnel's access to official information related to the COVID-19 epidemic will have a positive effect on alleviating negative emotions arising from the impact of the epidemic.

This study showed that psychological resilience was negatively correlated with all variables (*P* < 0.01), suggesting that better psychological resilience was associated with mitigating the extent of job burnout, depression, and anxiety in medical staff. In the hierarchical regression model, the Sobel test, the bootstrap test, and the structural equation model all confirmed the presence of a partial mediating effect of psychological resilience. Psychological resilience independently influenced the job burnout state and also alleviated the influence of anxiety and depression on job burnout. Therefore, we should pay attention to the positive impact of psychological resilience on the mental health of medical staff. Some studies have also shown that psychological resilience has mediating effects on negative emotions (23). The psychological resilience integrative model theory considers that psychological resilience mobilizes internal protective factors to resist an un-favourable external environment (24). Therefore, increased psychological resilience and the alleviation of negative emotions in medical personnel both have a significant positive effect on job burnout. Many studies have shown that mindfulness-based stress reduction methods can relieve negative emotional states in medical staff (25). Furthermore, social support also positively affects psychological resilience (26).

Therefore, extending better social support to medical staff members by paying attention to some important aspects of their lives, such as their family support, night shift status, and personal health, can relieve their stress and reduce job burnout. In addition, an emergency response medical team echelon should be established, and its members should be given long-term training in several areas, such as emergency knowledge, first aid skills, emotional control, and psychological counselling, to increase the psychological resilience of the emergency response team. Mental health courses, such as mindfulness-based stress reduction, can be added to the basic training curriculum of medical staff to improve their ability to respond to public health emergencies, such as COVID-19.

This study has some limitations. First, the samples in this study were selected using the convenience sampling method, and the sample source was limited to two tertiary hospitals; therefore, there were certain limitations. In addition, further analysis of medical staff at all levels is needed to understand the differences in the mental status of individuals of different healthcare professions to provide more precise theoretical support for targeted intervention measures.

## Conclusion

The COVID-19 epidemic will continue for some time, and medical personnel bear a heavy burden in the prevention and control of this disease. Considering that these personnel are under long-term work-related stress, various emotions interact to increase their psychological burden. The results of this study suggest that the psychological resilience of medical personnel plays a considerable role in regulating their mental health during their ongoing efforts to prevent and control the COVID-19 epidemic. Furthermore, anxiety and depression aggravate job burnout conditions in medical personnel. Therefore, future studies should focus on improving the psychological resilience of medical staff, with an emphasis on their various psychological states. Furthermore, their anxiety and depression symptoms should be actively addressed and relieved to prevent the aggravation of physical and mental illnesses due to the interaction of various negative emotions.

## Data Availability Statement

The original contributions presented in the study are included in the article/supplementary material, further inquiries can be directed to the corresponding author/s.

## Ethics Statement

The studies involving human participants were reviewed and approved by Ethics Committee of Zigong Fourth People's Hospital (201904). The patients/participants provided their written informed consent to participate in this study.

## Author Contributions

YC and LZ: writing – review, supervision, and project administration. WY: conceptualization, writing – original draft, writing – editing, and visualization. CN: methodology, writing – review, and editing. LY and HQ: investigation, resources, and supervision. PX: data curation, data analysis, and writing – review. All authors contributed to the article and approved the submitted version.

## Funding

Project of Sichuan Provincial Department of Science and Technology (22KJPX0156); Sichuan Research Center of Higher Vocational Education (GZY18C45); Medical Youth Innovative Program of Sichuan Province (Q21074); Research project of Zigong City Science & Technology and Intellectual Property Right Bureau (2021ZC22).

## Conflict of Interest

The authors declare that the research was conducted in the absence of any commercial or financial relationships that could be construed as a potential conflict of interest.

## Publisher's Note

All claims expressed in this article are solely those of the authors and do not necessarily represent those of their affiliated organizations, or those of the publisher, the editors and the reviewers. Any product that may be evaluated in this article, or claim that may be made by its manufacturer, is not guaranteed or endorsed by the publisher.
